# GGNBP2 is necessary for testis morphology and sperm development

**DOI:** 10.1038/s41598-017-03193-y

**Published:** 2017-06-07

**Authors:** Anqi Chen, Jixi Li, Lesheng Song, Chaoneng Ji, Marion Böing, Jinzhong Chen, Beate Brand-Saberi

**Affiliations:** 1 State Key Laboratory of Genetic Engineering, Institute of Genetics, School of Life Sciences, Fudan University, Shanghai, 200433 P.R. China; 2 Department of Anatomy and Molecular Embryology, Ruhr-University Bochum, Bochum, Germany

## Abstract

Gametogenetin Binding Protein 2 (GGNBP2) was identified as a tumor suppressor and verified as such by several studies. GGNBP2 has also been reported to be essential for pregnancy maintenance via regulation of trophoblast stem cells. Gametogenetin (GGN) is a testicular germ cell-specific gene expressed in adult testes. As a potential GGN1-interacting protein, the role of GGNBP2 in spermatogenesis has not yet been clarified. We generated heterozygous GGNBP2 knockout mice and bred them by intercrossing. We found that among the offspring, homozygous GGNBP2 knockout (KO) mice were present in severely reduced numbers. The GGNBP2 KO pups developed normally, but the male siblings showed dramatically reduced fertility. In these male homozygous GGNBP2 KO mice, the only pathological finding was abnormal morphology of the testes and absence of spermatozoa. In addition, increased apoptosis was observed in the testes of GGNBP2 KO mice. SOX9 staining revealed that SOX9-positive Sertoli cells were absent in the seminiferous tubules. In homozygous mice, proliferating cell nuclear antigen (PCNA)-positive cells were localized in the lumen of the convoluted seminiferous tubules. These results suggest that GGNBP2 plays a key role in spermatogenesis by affecting the morphology and function of SOX9-positive Sertoli cells.

## Introduction

ZNF403, a C3HC4-type zinc finger protein, was initially identified as a laryngeal carcinoma-related protein 1 (LCRG1) by the DD-PCR analysis of the tumor suppressor locus D17S800-D17S930 expression tags in laryngeal carcinoma cells^1^. GGN is a testicular germ cell-specific gene expressed in the adult testis from late pachytene spermatocytes to round spermatids. A yeast two-hybrid screen identified ZNF403 and GGNBP1 as potential interaction partners of GGN1^2^. Thus, ZNF403 was named Gametogenetin-binding protein 2 (GGNBP2) after GGNBP1. It has been previously reported that knockdown of ZNF403 inhibits cell proliferation and induces G2/M arrest^3^. GGNBP2, also named DIF-3 (dioxin inducible factor-3), is a target gene that mediates the reproductive toxicity induced by the environmental toxic agent dioxin. Thus, it is referred as a dioxin-induced nuclear factor and functions in spermatogenesis and cell differentiation^4^.

The newly described 17q12 microdeletion syndrome has been associated with MODY5 (maturity-onset of diabetes of the young type 5), cystic renal disease, pancreatic atrophy, liver abnormalities, cognitive impairment and structural brain abnormalities, and a congenital diaphragmatic hernia (CDH)^5, 6^. Deletion of the 17q12 region results in haploinsufficiency of 17 genes, including GGNBP2.

Recently, a study reported that down-regulated expression of GGNBP2 is associated with drug resistance in ovarian cancer^7^. Lan^8^ reported that GGNBP2 was a novel breast cancer tumor suppressor functioning as a nuclear receptor corepressor to inhibit ERα activity and tumorigenesis. During the writing of this manuscript, GGNBP2 was reported to be essential for pregnancy maintenance via regulation of mouse trophoblast stem cell proliferation and differentiation. GGNBP2 null mutant embryos died in utero between embryonic days 13.5 to 15.5 with dysmorphic placenta, which was characterized by excessive nonvascular cell nests consisting of proliferative trophoblastic tissue and abundant trophoblast stem cells (TSCs) in the labyrinth^9^.

Here, we confirm that the majority of GGNBP2 KO mice die in utero and that only a few mice survive to birth and reach adulthood. Furthermore, GGNBP2 KO males are infertile and show extensive defects in spermatogenesis.

## Results

### Absence of GGNBP2 results in infertility in mice

**Figure 1:**
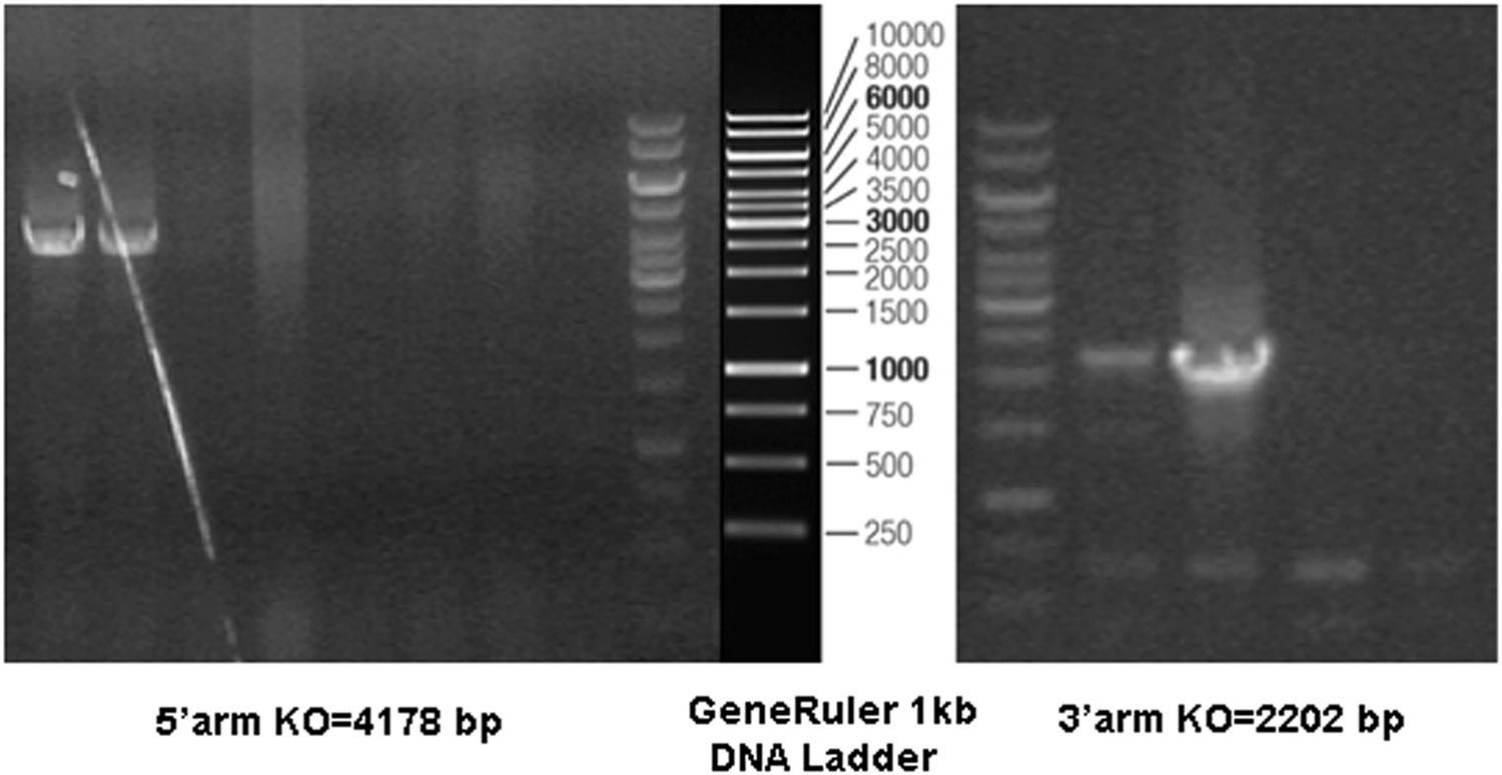
Identification of gene targeted ES cells. Ganciclovir resistance targeted ES clones were identified by PCR with the Neo-specific vector primer (P2, P3) and host genome-specific primers (P1 for GGNBP2 exon 3-specific, P4 for GGNBP2 intron 7-specific). Targeted clones were expected to produce both 4178 bp 5′ arm amplicon and 2202 bp 3′ arm amplicon. Only two clones were dual positive. The GeneRuler 1 kb DNA Ladder is a DNA Marker, and the numbers and bands between the two agarose gels were provided by the manufacturer (Thermo Scientific, #SM0312).

The GGNBP2 target vector, pBR322-MCS-2S-GGNBP2, was confirmed by sequencing. In total, 96 ganciclovir resistance-targeted ES clones were further identified using PCR. Two clones were positive, and both clones carried the 5′ arm and the 3′ arm (Fig. 1). The targeted ES cells were injected into 87 blastocysts and then implanted into 6 uteri of female mice for development. Seven chimeric male mice were generated and backcrossed into C57BL/6 background female mice. Eight heterozygous offspring were verified using the method described above.

Heterozygous offspring were interbred, and 179 pups of twenty breedings were used to analyze the phenotypes of three different genotypes; wt/wt, wt/ko and ko/ko (Table 1). Fifty-five animals had the wt/wt genotype and 120 had the wt/ko genotype. The number of ko/ko mice was 4 (3 males and 1 female), which was much less than expected. There was no obvious difference in the weight or activities between the genotypes, except that all male KO mice were infertile.

**Table 1:** The genotype distribution of the pups resulting from heterozygous offspring interbreeding.

Sex	Male	Female	Total
genotype			
wt/wt	20	35	55
wt/ko	68	52	120
ko/ko	3	1	4
			179

### Absence of GGNBP2 results in abnormal testis morphology

**Figure 2:**
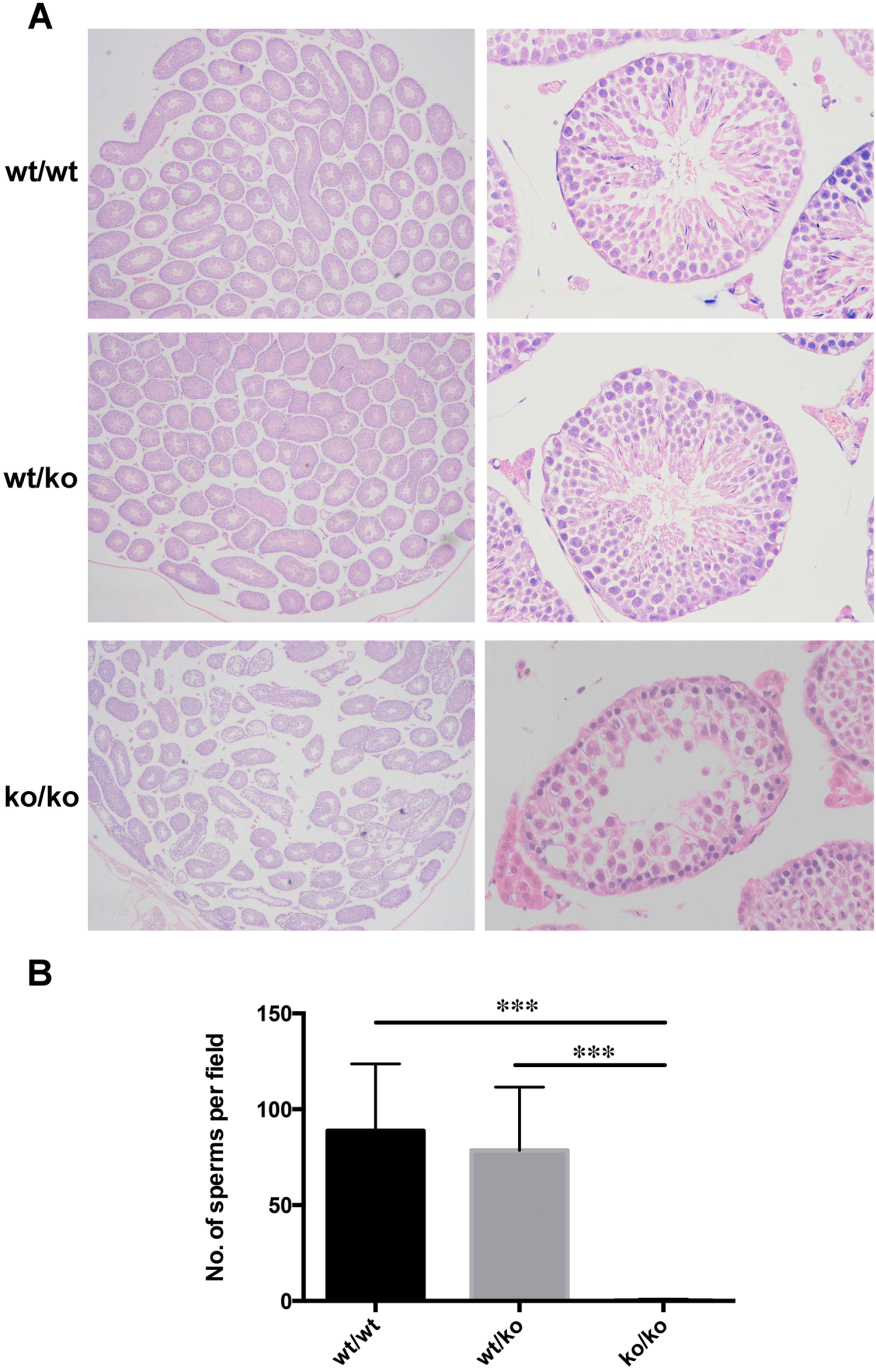
Absence of GGNBP2 results in abnormal testis morphology. (**A**) HE testis histology of the three murine genotypes. HE sections were produced using the standard method. From top to bottom are the testes of wild-type, heterozygous and GGNBP2 KO mice. The left row is 10 × 10 under LM and the right row is 10 × 40 under LM. (**B**) Quantification of the number of sperm per field (field of view at 400x magnification) in the intersecting surface of the testes. The graph shows the mean ±  SEM of 5 independent fields. Statistical analysis was performed using Student’s t-test. ***P < 0.001.

Male and female mice with genotypes of wt/wt, wt/ko and ko/ko were sacrificed and the corresponding phenotypes were determined with routine histological analysis. The only identified abnormality was found in the testes of male homozygous mice (ko/ko), in which the morphology of the convoluted seminiferous tubules was visibly altered. A histological analysis of the epithelium of the convoluted seminiferous tubules revealed that these appeared lower and more loosely structured than in the wild-type and heterozygous siblings (Fig. 2A). There was no significant difference in the number of sperm between the wild-type and heterozygous mice. However, nearly no sperm cells could be observed inside the seminiferous tubules of homozygous KO mice. The number of sperm in wild-type, heterozygous and GGNBP2 KO mice were 88.80 ± 15.61 sperm/field, 78.60 ± 14.76 sperm/field and 0.40 ± 0.24 sperm/field (field of view at 400x magnification), respectively. The P values of ko/ko to wt/wt and of ko/ko to wt/ko were less than 0.001, and the P value of wt/wt to wt/ko was 0.648 (Fig. 2B).

### Absence of GGNBP2 increases apoptosis in the testis

**Figure 3:**
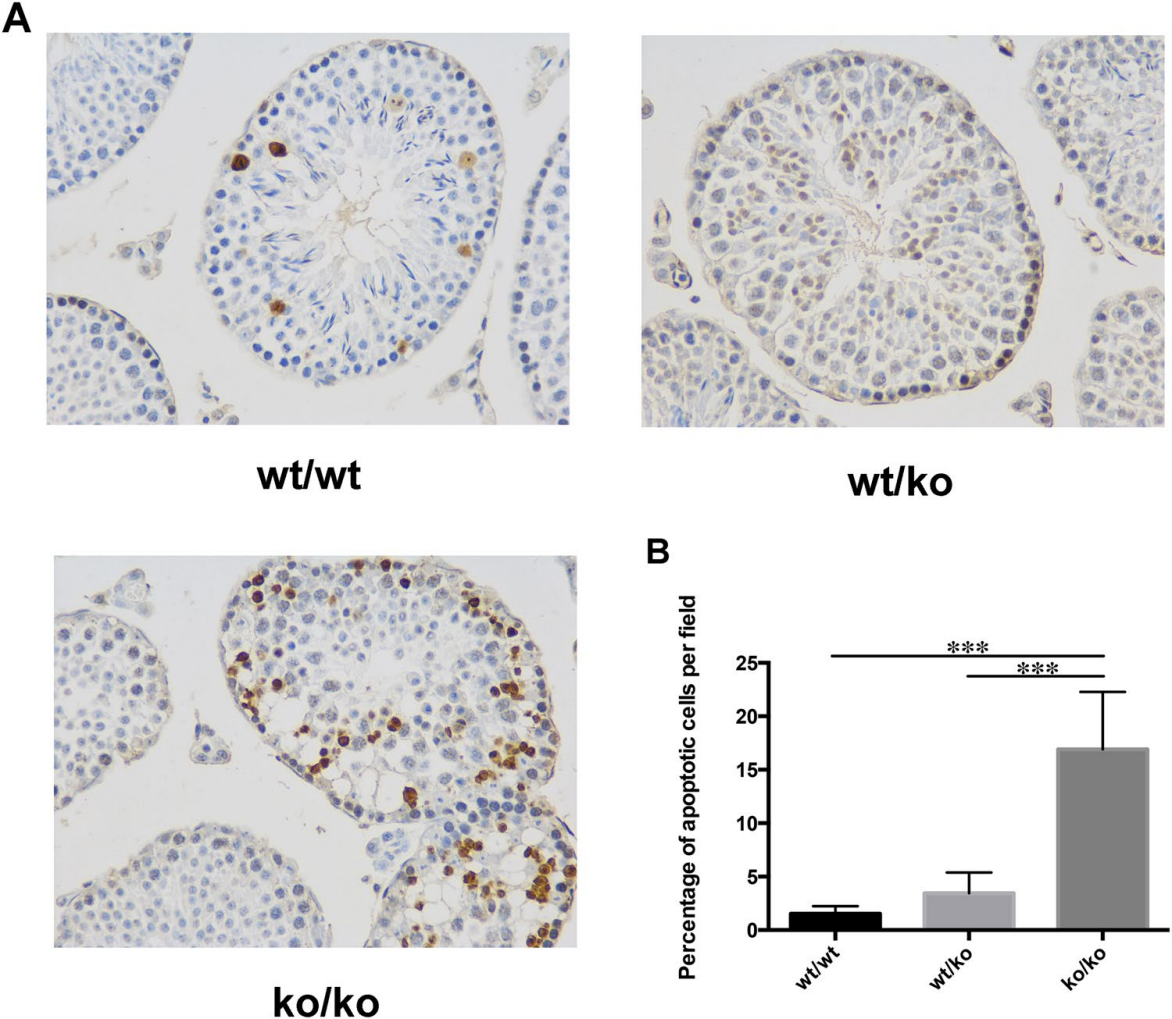
Absence of GGNBP2 increases apoptosis in testis. (**A**) Apoptosis analysis of the three murine genotypes. From top to bottom are the testis of wild-type, heterozygous and GGNBP2 KO mice. 10 × 40, LM. (**B**) Quantification of the percentage of apoptotic cells per field (field of view at 400x magnification) in the intersecting surface of the testes. The graph indicates the mean ± SEM of 5 independent fields. Statistical analysis was performed using Student’s t-test. ***P < 0.001.

To identify the cause of infertility, a TUNEL-based kit was used to detect the apoptosis of mouse testis. There were more apoptotic cells in the testis of GGNBP2 KO mice. The apoptotic cells in wild-type mice and heterozygous mice were located near the basement membrane. In contrast, apoptotic cells in the testes of GGNBP2 KO mice were more dispersed towards the lumen of the tubules (Fig. 3A). The percentage of apoptotic cells per field (field of view at 400x magnification) in wild-type, heterozygous and GGNBP2 KO mice were 1.54% ± 0.70%, 3.46% ± 1.93% and 16.91% ± 5.37%, respectively. The P values of ko/ko to wt/wt and of ko/ko to wt/ko were less than 0.001, and the P value of wt/wt to wt/ko was 0.069 (Fig. 3B). We also detected apoptotic cells in the stomach and small intestine. However, there was no detectable significant difference among the three genotypes in these organs (data not shown).

### Effect of GGNBP2 deletion on proliferation in spermatogonia

**Figure 4:**
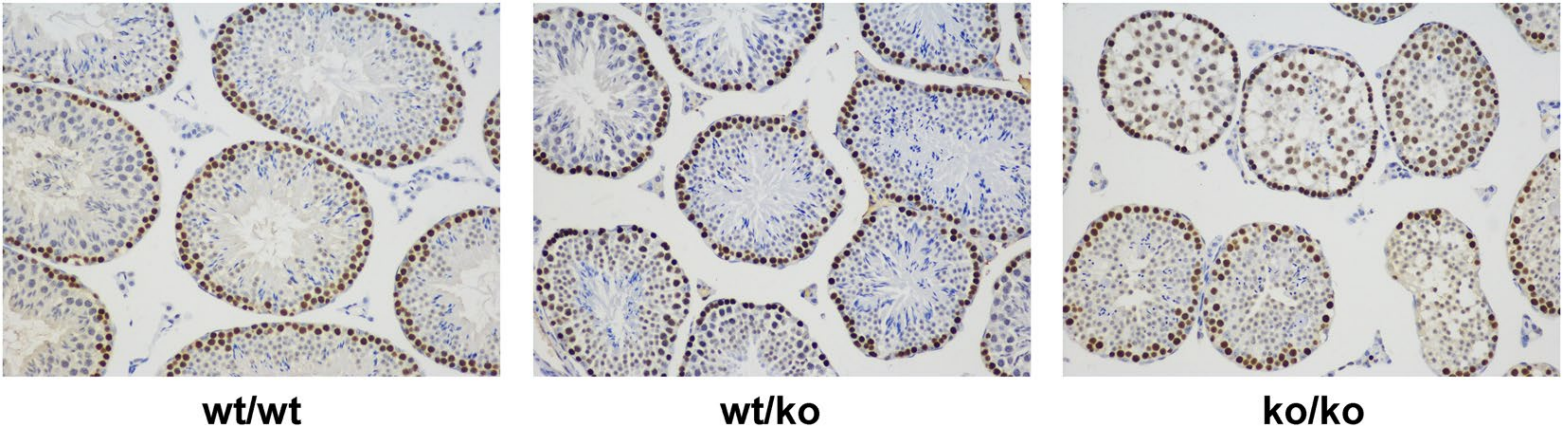
PCNA IHC of the testes of the three murine genotypes. From the left to the right is the testis of wild-type, heterozygous and GGNBP2 KO mice. 10 × 40, LM.

In normal adult testes, spermatogonia undergo proliferation. PCNA is an indicator of cells during DNA synthesis. To compare spermatogonia proliferation in the three genotypes, we used PCNA-specific antibodies to visualize the cells undergoing DNA synthesis. In wild-type testes, strong PCNA positivity was detected in the spermatogonia, suggesting active cell proliferation. The PCNA-positive spermatogonia were located near the basement membrane and were arranged regularly in wild-type mice. The PCNA signal of other cells was barely above background level in wild-type testis. The same was also found in heterozygous testis. In mice with the ko/ko genotype, numerous PCNA-positive cells were dispersed throughout many of the tubules; in other tubules, they were localized in the basal compartment (Fig. 4).

### Absence of GGNBP2 results in impaired SOX9 expression in the testis

**Figure 5:**
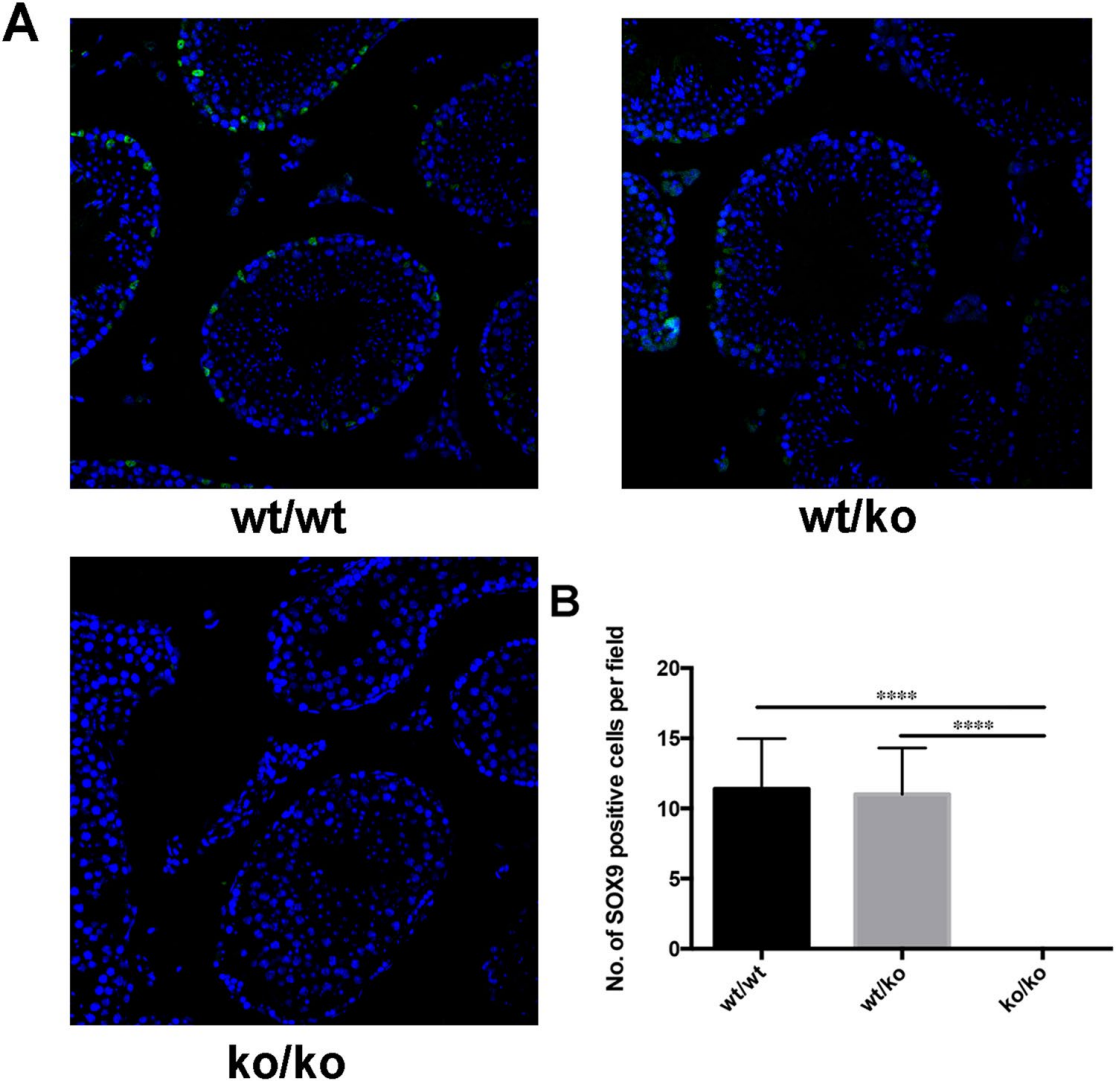
Absence of GGNBP2 results in impaired SOX9 expression in the testis. (**A**) SOX9 expression pattern of the testes of the three murine genotypes. From the left to the right are the testis of wild-type, heterozygous and GGNBP2 KO mice. 10 × 40, LM. (**B**) Quantification of the number of SOX9-positive cells per field (field of view at 400x magnification) in the intersecting surface of the testes. The graph shows the mean ± SEM of 5 independent fields. Statistical analysis was performed using Student’s t-test. ****P < 0.0001.

SOX9 plays a key role in promoting the development of testis cords, maturation of Sertoli cells and the maintenance of spermatogenesis in adult testis^10, 11^, and it is a useful marker in the identification of Sertoli cells^12^. Signals were found regularly in the testes of both wild-type mice and heterozygous mice. In contrast, no Sox9 staining was present in the contorted seminiferous tubules of homozygous KO mice (Fig. 5A). The number of Sox9-positive cells in wild-type, heterozygous and GGNBP2 KO mice were 11.40 ± 1.60 cells/field, 11.00 ± 1.48 cells/field and 0.00 ± 0.00 cells/field (field of view at 400x magnification), respectively. The P values of ko/ko to wt/wt and ko/ko to wt/ko were less than 0.0001, and the P value of wt/wt to wt/ko was 0.859 (Fig. 5B).

## Discussion

To determine the function of GGNBP2, we established GGNBP2 heterozygous KO mice. GGNBP2 heterozygous KO female and male mice were paired, and the heterozygous offspring were approximately twice the number of wild-type mice (Table 1), which was expected according to Mendelian laws. However, the ratio of homozygous KO mice was much less than expected. Only four GGNBP2 KO mice were obtained from a total of 179 offspring. This finding suggests that something unexpected occurred to the fetuses with the ko/ko genotype. Recently, work by Li revealed that the death of GGNBP2 null embryos is caused by insufficient placental perfusion as a result of a remarkable decrease in both fetal and maternal blood vessels in the labyrinth^9^. The labyrinth plays a role in material interchange between the mother and fetus, which supports the development of the fetus^13^. The labyrinth may be responsible for the unusually low rate of KO mice. Although we observed an extremely low number of fetuses with the ko/ko genotype, these mice still had a chance of being born. This might be caused by compromised placental perfusion.

Heterozygotes of GGNBP2 were normal both in terms of phenotype and fertility. As expected, the number of heterozygotes (120) was nearly doubled when compared with that of the wild-type mice (55). This finding suggested that heterozygote genotype presented no disadvantages to the wild-type mice, and thus one functional allele was sufficient to compensate for the absence of the other. Histological analysis also showed no significant differences between wild-type and heterozygote mice (Fig. 2A). The only bias against homozygous GGNBP2 KO offspring could be explained by a report by Li^9^. Although there was no obviously abnormal morphology in GGNBP2 KO mice, none of them could bear any offspring even when inbred with wild-type mice. The only positive pathological finding was primary male infertility. Convoluted seminiferous tubules had significantly disarranged morphology, and there were much less sperm cells observed in the testes of KO mice (Fig. 2B). This result explains why GGNBP2 KO mice present with male infertility.

Azoospermy was found in GGNBP2 KO mice. From the histological sections, only a few sperm could be occasionally detected in the intersecting surface of the testes in KO mice. Moreover, the convoluted seminiferous tubules lost their morphology, the epithelium exhibited distortion, and the cells were scattered in the tubules. A TUNEL-based kit was employed to identify the cause of the azoospermy. The testis of GGNBP2 KO mice presented with much more apoptotic cells than that of wild-type or heterozygous mice (Fig. 3B). Apoptosis was accompanied by morphological changes. This result suggests that the almost complete absence of spermatozoa and azoospermy as judged from flushing the epididymis might be the result of increased apoptosis.

Mitosis of spermatogonia is important for maintaining the homeostasis of the testis and the development of sperm. Spermatogonia are normally localized closely to the basement membrane. A subpopulation of spermatogonia is capable of undergoing mitosis to maintain the homeostasis of spermatogonia during spermatogenesis, while other spermatogonia mature into primary spermatocytes. PCNA plays an essential role in DNA replication, synthesis, repair, and cell cycle control, and it has proven to be a useful indicator of the cells involved in DNA synthesis and repair^14^. PCNA is detectable in mitotically proliferating spermatogonia, but not in spermatocytes, which have just entered meiosis. PCNA staining was observed in spermatogenic cells in later stages of meiotic prophase, specifically, zygotene and pachytene spermatocytes undergoing meiotic recombination. It reflects a function of DNA excision repair in meiosis^14, 15^. The distribution of PCNA-positive cells gives further clues to the testis abnormality in GGNBP2 KO mice (Fig. 4). Two potential scenarios might result in PCNA-positive cells to shift from the location of the basal compartment towards the adluminal compartment. However, both compartments are ill-defined due to a compromised structure of the morphologically altered Sertoli-cells. One could be the failure of entrance into meiosis of spermatogonia, in which the cells stay in mitosis and continue to express PCNA. The other scenario could be that spermatogonia lose their support from Sertoli cells, which results in a shift towards the tubule lumen. In the latter case, the key event might be a functional disturbance in the Sertoli cells.

SOX9 is a transcription factor associated with Sertoli cell differentiation^10^, and it is activated by testis-determining factor binding to its promoter^16^. Next, SOX9 activates the transcription of the anti-Müllerian hormone gene of Sertoli cells and results in the development of male sexual organs^10^. SOX9 is a reliable marker in the identification of Sertoli cells^11, 12, 17^. The expression pattern of SOX9 in wild-type and heterozygous mice were nearly the same as that described in normal adult testis (Fig. 5B). The signals located in the nucleus of Sertoli cells are normally close to the basement membrane. However, no SOX9-positive Sertoli cells were identified in the testis of GGNBP2 KO mice (Fig. 5A). Thus, we inferred that the loss of SOX9-positive Sertoli cells might result in the morphological changes observed in the testes of homozygous GGNBP2 KO mice, causing azoospermy due to severely compromised spermatogenesis. SOX9 has been reported to be essential for maintaining adult fertility^18^, which is consistent with our results. However, the exact molecular mechanism of GGNBP2-related azoospermy requires further study.

## Materials and Methods

### Establishment of GGNBP2 KO mice

**Figure 6:**
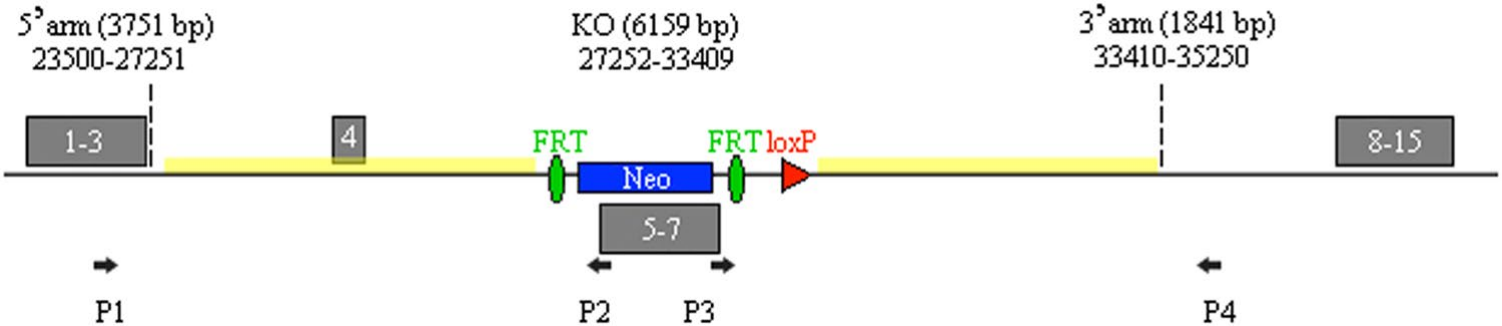
Schematic of the GGNBP2 KO targeting vector. The GGNBP2 KO vector was designed to replace GGNBP2 from exon 5 to exon 7 (6159 bp, from bp 27252 to bp 33409 of ENSMUST00000100685) with a Neo expression fragment that results in the destruction of all potential open reading frames of GGNBP2 expression isoforms. The numbers in the gray shadows are the exon numbers of GGNBP2. Two homologous arms (3751 bp 5′ arm from bp 23500 to bp 27251 of ENSMUST00000100685, 1841 bp 3′ arm from bp 33410 to bp 35250 of ENSMUST00000100685) flank the Neo expression frame. One loxP and two FRT sites are located near the terminus of the Neo expression frame. P1 (GGNBP2 specific), P2 (Neo specific), P3 (Neo specific) and P4 (GGNBP2 specific) were the primers used to amplify the targeted genome.

The targeting vector for GGNBP2 KO was constructed to delete exons 5 to 7 using a similar method as described previously^19, 20^. The Neo expression frame was inserted into intron 4 and intron 7, and the length of the two flanking arms were 3751 bp and 1814 bp, respectively. This produced a frame-shift of all seven putative expression isoforms of GGNBP2, and no functional protein could be produced (Fig. 6).

The SCR012 ES cells came from a 129SV/EV male embryo. The ES cells targeting method was described by Joyner^21^. Ganciclovir resistance targeted clones were further confirmed by PCR. The 5′ arm was amplified using the GGNBP2-specific primer ATGGTGATGGAATTTCCTGAC and the Neo-specific primer GGCCTACCCGCTTCCATTGCTC, and the expected fragment had a length of 4178 bp. The 3′ arm was amplified with the Neo-specific primer CCGTGCCTTCCTTGACCCTGG and the GGNBP2-specific primer CCATGGCAATACTGATGATAGCC, and the expected fragment had a length of 2202 bp.

Correctly targeted ES cells were injected into C57BL/6 blastocysts, and chimeric mice were generated. Experimentally produced chimeric mice were obtained according to the method described by Nagy^22^. Chimeric male mice were backcrossed with C57BL/6 background female mice to produce GGNBP2 wt/ko founder mice. The GGNBP2 wt/ko founders were used to produce the F2 mice with genotypes of wt/wt, wt/ko and ko/ko. The genotype was identified using the method described above.

All animal procedures were performed in accordance with institutional guidelines under protocols approved by the Animal Care and Use Committee of Fudan University.

### Histology of GGNBP2 KO mice

The F2 heterozygous mice were used to produce F3 offspring. The number, growth and development of F3 offspring with different genotypes were analyzed. The present individuals were sacrificed and analyzed using HE staining.

### Immunohistochemistry analysis

Immunohistochemistry (IHC) analyses were employed to reveal the potential mechanism of male infertility and abnormality of spermatogenesis in GGNBP2 KO mice. A rabbit anti-PCNA antibody (AB18197, Abcam) and HRP-conjugated goat anti-rabbit IgG (AB182016, Abcam) were used to identify the DNA replication-related changes in the testis cells. The signals were visualized using a DAB Substrate Kit (ab64238, Abcam).

A rabbit anti-Sox9 antibody (AB5535, Millipore) and a goat anti-rabbit IgG-Alexa488 (ab150077, Abcam) were used to identify Sertoli cells, the cell nuclei were stained with DAPI.

### Apoptosis analysis

A TUNEL (TdT-mediated dUTP nick end labeling)-based kit (Roche) was used to detect the apoptosis of cells in the mice testes according to the manufacturer’s protocol. The DAB agent was used to visualize the data collected using a Nikon DS-U3 system and analyzed using Image-Pro Plus6.0 software. Five independent fields of vision were chosen as representative fields in each mouse genotype.

### Statistical analysis

The data were analyzed using Prism 6 software, and all statistical analyses were performed using Student’s t-test. Significance was defined as P < 0.05.

## References

[1] Li Y, Chen Z (2004). Molecular cloning and characterization of LCRG1 a novel gene localized to the tumor suppressor locus D17S800-D17S930. Cancer letters.

[2] Zhang J (2005). Yeast two-hybrid screens imply that GGNBP1, GGNBP2 and OAZ3 are potential interaction partners of testicular germ cell-specific protein GGN1. FEBS letters.

[3] Guan R (2012). Knockdown of ZNF403 inhibits cell proliferation and induces G2/M arrest by modulating cell-cycle mediators. Molecular and cellular biochemistry.

[4] Ohbayashi T (2001). Dioxin induces a novel nuclear factor, DIF-3, that is implicated in spermatogenesis. FEBS letters.

[5] Hendrix NW, Clemens M, Canavan TP, Surti U, Rajkovic A (2012). Prenatally diagnosed 17q12 microdeletion syndrome with a novel association with congenital diaphragmatic hernia. Fetal diagnosis and therapy.

[6] Pasmant E (2008). Characterization of a 7.6-Mb germline deletion encompassing the NF1 locus and about a hundred genes in an NF1 contiguous gene syndrome patient. European journal of human genetics: EJHG.

[7] Yin F (2014). Downregulation of tumor suppressor gene ribonuclease T2 and gametogenetin binding protein 2 is associated with drug resistance in ovarian cancer. Oncology reports.

[8] Lan ZJ (2016). GGNBP2 acts as a tumor suppressor by inhibiting estrogen receptor alpha activity in breast cancer cells. Breast cancer research and treatment.

[9] Li S (2016). Ggnbp2 Is Essential for Pregnancy Success via Regulation of Mouse Trophoblast Stem Cell Proliferation and Differentiation. Biology of reproduction.

[10] De Santa Barbara P (1998). Direct interaction of SRY-related protein SOX9 and steroidogenic factor 1 regulates transcription of the human anti-Mullerian hormone gene. Molecular and cellular biology.

[11] Banco B (2016). Immunohistochemical expression of SOX9 protein in immature, mature, and neoplastic canine Sertoli cells. Theriogenology.

[12] Hemendinger RA, Gores P, Blacksten L, Harley V, Halberstadt C (2002). Identification of a specific Sertoli cell marker, Sox9, for use in transplantation. Cell transplantation.

[13] Simmons DG (2008). Early patterning of the chorion leads to the trilaminar trophoblast cell structure in the placental labyrinth. Development (Cambridge, England).

[14] Hall PA (1990). Proliferating cell nuclear antigen (PCNA) immunolocalization in paraffin sections: an index of cell proliferation with evidence of deregulated expression in some neoplasms. The Journal of pathology.

[15] Chapman DL, Wolgemuth DJ (1994). Expression of proliferating cell nuclear antigen in the mouse germ line and surrounding somatic cells suggests both proliferation-dependent and -independent modes of function. The International journal of developmental biology.

[16] Moniot B (2009). The PGD2 pathway, independently of FGF9, amplifies SOX9 activity in Sertoli cells during male sexual differentiation. Development (Cambridge, England).

[17] Chen SR, Liu YX (2016). Testis Cord Maintenance in Mouse Embryos: Genes and Signaling. Biology of reproduction.

[18] Barrionuevo F (2009). Testis cord differentiation after the sex determination stage is independent of Sox9 but fails in the combined absence of Sox9 and Sox8. Developmental biology.

[19] Liu P, Jenkins NA, Copeland NG (2003). A highly efficient recombineering-based method for generating conditional knockout mutations. Genome research.

[20] Chan W (2007). A recombineering based approach for high-throughput conditional knockout targeting vector construction. Nucleic acids research.

[21] AL, J. *Gene Targeting* Oxford University Press (1994).

[22] Nagy, A., Gersenstein, M., Vinteraten, K. *et al*. *Manipulating the Mouse Embryo: A laboratory Manual* New York: Cold Spring Harbor Laboratory Press **469**, 198, 487 (2003).

